# A dataset of urban traffic flow for 13 Romanian cities amid lockdown and after ease of COVID19 related restrictions

**DOI:** 10.1016/j.dib.2020.106318

**Published:** 2020-09-17

**Authors:** Alexandru Iovanovici, Dacian Avramoni, Lucian Prodan

**Affiliations:** Department of Computers and Information Technology, Politehnica University Timisoara, Bld. Vasile Pirvan, nr. 2, 300585 Timisoara, Romania

**Keywords:** Intelligent transportation systems, Traffic flow, Micro-simulation, COVID19, Timisoara, Romania

## Abstract

This dataset comprises street-level traces of traffic flow as reported by Here Maps™ for 13 cities of Romania from 15th. of May 2020 and until 5th. of June 2020. This covers the time two days before lifting of the mobility restrictions imposed by the COVID19 nation-wide State of Emergency and until four days after the second wave of relaxation, announced for 1st. of June 2020. Data were sampled at a 15-min interval, consistent with the Here API update time. The data are annotated with relevant political decisions and religious events which might influence the traffic flow. Considering the relative scarcity of real-life traffic data, one can use this data set for micro-simulation during development and validation of Intelligent Transportation Solutions (ITS) algorithms while another facet would be in the area of social and political sciences when discussing the effectiveness and impact of statewide restriction during the COVID19 pandemic.

## Specifications Table

**Table:** 

Subject	Transportation
Specific subject area	Traffic flow demand data
Type of data	Table Figure CVS data files
How data were acquired	Software application (available in the dataset, as part of the article), developed using Python language, using Here API for gathering raw data regarding live traffic and a set of custom developed scripts for cleaning the data (detailed below) and plotting visual representations of the instantaneous traffic flow. Hand annotation was used for providing supplementary data and information for specific events regarding the national policy against COVID19 and also for description of the cities
Data format	Raw Preprocessed Annotated
Parameters for data collection	The datasets covers the period from 15th. of May 2020 and until 5th. of June 2020, with a sampling period of 15 minutes, using the standard Here Maps Traffic API
Description of data collection	There are 3 software scripts used: one is responsible for job automation and runs the grabbing script at a 15 minutes interval, which subsequently launches the API requests for each of the cities and writes the XML files with raw data on drive. Later the third script iterates over the XML files and extracts the road information data and traffic flow data, discarding the geometrical properties of the road.
Data source location	Politehnica University Timisoara Department of Computers and Information Technology, Advanced Computing Systems and Architectures Laboratory 2nd. Vasile Pirvan, 300585 Timisoara, Romania 45.7439041,21.2255296 Available online https://data.mendeley.com/datasets/g64s8h9k57
Data accessibility	Repository name: Mendeley Data Data identification number: g64s8h9k57 Direct URL to data: https://data.mendeley.com/datasets/g64s8h9k57

## Value of the Data

• There is a scarcity of data available regarding traffic flow and road use demand. Even if larger cities in highly developed nations have near real-time data from ITS systems, in other cases those data are practically impossible to gather with good quality and at decent costs. This data set covers a broad range of demands and loads, form almost empty roads (during COVID19 restrictions) and up to full traffic (after second set of relaxation rules);
• This dataset is directly useful for practitioners in the field of ITS systems design, for assessing transportation capacity and developing algorithms and policies for congestion prediction and mitigation and also for sociologists doing research regarding the impact of COVID19 restrictions and the reaction of the public to the restrictions and gradual lifting of the restrictions.
• The main usage of the data, in the field of ITS, is to provide real-life data from a variety of Romanian cities (ranging from small to large in population, area and road network size) useful for training machine learning algorithms for prediction of congestion and for simulation of the impact of traffic incidents over the traffic flow. Practitioners in the field of social sciences can benefit from the data in the analysis of specific reactions of the population to COVID19 restrictions.
• Descriptive statistics could be used for simple analysis of data and detection of anomalies in the traffic flow which in turn can be used for inferring hidden events such as an incident on a minor street which feeds to a major artery.
• Machine learning methods and tools can be used for identifying signature-features of traffic flow which predict congestion, with high spatial resolution.
• Qualitative analysis of the impact of COVID19 transportation restrictions can be made, with ramification of both the economic sector and epidemiological one

## 1 Data Description

In the field of Transportation there is a distinct subfield of Intelligent Transportation Systems (ITS) characterized by the usage of methods and tools of computation, mathematics and control theory for deriving means of maximizing the usability of the existing infrastructure (transportation capacity and quality) or the decision to develop new infrastructure [1]. One of the current important topics in this field is related to congestion prediction [2,3], while a lot of the approaches rely on the means and methods of machine learning to leverage the value of the past (historic data) in order to predict the future (when congestion will arise) [4]. Another subject of interest, directly connected to the problem of congestion is the one related to the traffic incident management [2,5]. A lot of the rules, policies and the systems are designed and work well in stable nominal conditions (when all the participants obey the traffic laws and everything works as intended). Analysis done over the root cause of major gridlocks showed that the complex dynamics involved with road traffic allows minor incidents (i.e. a car not giving way when changing lanes) to become major sources of trouble spanning dozens of minutes a few blocks (hundreds of meters) radius [6].

The resolution of both problems can be addressed in a virtual environment using what is called traffic micro-simulation [7]. When fed high quality data and with a good description of the existing infrastructure, current software tools for microsimulation are capable of mirroring actual traffic conditions over a time-span ranging from dozens of minutes to hours [2]. Topology of the road infrastructure and the placement of road signs and traffic signaling plans are core components of the simulation scenarios and can be obtained either from local authorities or from open data ([4,8]) and an initial leg-work (for collecting data regarding signaling plans). The missing component is represented by the actual conditions on the road, which can be obtained by the existing infrastructure (car counting loops and equipment) - which is costly to deploy and provide low spatial resolution - or by deploying human observers for making assessments - which is costly and provides low temporal resolution [2,4].

Over the last decades, with the development of mobile applications targeted at assisting drivers on the road, a new set of sources has appeared in the form of traces form mobile devices of the drivers (or passengers), but still most of them are not providing means of accessing historical data [9]. Major players in the field provide current data inside their applications and most of the time historic data are provided in an aggregate manner, which suffice for the average user, but are not of good enough quality for the practitioners in the field of ITS [10,11].

We selected Here Maps (™) [10] for gathering data because they provide data access via API, allowing scripted automation, and the collection of the data in an automated manner is allowed by their Terms and Conditions. Data provided by the API is always for current conditions but can be inferred by the Here Maps engine when the actual number of participants to the traffic is low, expressed by Confidence Level (see below) [10]. We have chosen a sampling frequency of 4 times per hour (once every 15 min) based on empirical observations regarding when data changes and limitations in the software license we used. A smaller than 5 min sampling period is not useful because the Here Traffic API does not update the data that often.

Each of the cities was defined through a rectangular bounding box with geo-coordinates described in Table 1.

**Table 1:** List of cities comprising the dataset, the coordinated of the corresponding bonding-boxes and some remarks over the inclusions of these cities in the set.

	Bounding box coordinates				
City	Top-Left Lat.	Top-Left Lon.	Bottom-right Lat.	Bottom-right Lon.	Comments
Timișoara	45.8162	21.1324	45.6023	21.3141	third largest city in Romania, westernmost in the dataset
Cluj-Napoca	46.8028	23.5361	46.7512	23.6915	second-largest city in Romania
Arad	46.2212	21.2611	46.1296	21.3840	mid-sized city close to the border with Hungary, part of the Timisoara metro-area
Oradea	47.0952	21.8962	46.9948	22.0066	mid-sized city, en-route from Hungarian border towards Cluj and Transilvania area
Caransebes	45.4456	22.1951	45.3671	22.2511	mid-sized city en-route E70 road; has city belt
Lugoj	45.7003	21.8923	45.6681	21.9359	small city en-route E70;
Craiova	44.3536	23.7688	44.2658	23.8795	Mid-sized city in Southern part of Romania
Constanta	44.2300	28.5921	44.1160	28.6627	large city; seaport at the Black Sea
Ploiesti	44.9906	25.9795	44.8849	26.1112	large city, close the capital Bucharest and en-route to central Romania
Bacau	46.6159	26.8739	46.4939	26.9669	close to Suceava
Suceava	47.7163	26.1551	47.5921	26.3574	most cases of COVID19 in Romania; under military lockdown for Suceava and surrounding area
Brasov	45.7174	25.5268	45.6061	25.6878	old mid-sized city in central part of Romania
Sibiu	45.8486	24.0865	45.7401	24.2223	old mid-sized city in central part of Romania

The time span covered by this dataset ranges from 15th. of May 2020 and until 5th. of June, during the mobility restrictions imposed by Romanian authorities for containing the COVID19 pandemic and provides the opportunity for capturing a diverse and broad spectrum of scenarios in terms of traffic demand data. The cities for which we provide traffic data, also represent a diverse set in terms of demographics, urban development and geographical placement in Romania. A detailed description is provided in Table 1.

The dataset consists of three parts:

1. CSV files with traffic flow data for each of the monitored cities: these are stored under the ./flow folder and follow the **<cityName>.csv** naming structure, where cityName is one of the cities from Table 1 (i.e. **./flow/timisoara.csv**). Each line represents a single record and the fields are presented in the header of the file, in the following order: **cityName, date, time, de, le, pc, qd, ff, cn, jf, sp, su, ty.** The naming of the fields follows the notation presented in Table 2.

**Table 2:** List of parameters gathered from the Here Maps API regarding traffic flow and comments over the data.

Parameter	Meaning	Comments
**de**	Text description of the road	Usually the street name or road designation on highways
**le**	Length of the road segment	In this dataset unit is kilometer
**pc**	Point Traffic Message Channel (TMC) Location Code	According to ISO 14819-3
**qd**	Queueing direction for the cars waiting/stalled on the specific road segment: + or −	When traffic becomes congested, a queue of vehicles begins to build up in the opposite direction to the driving direction. The value “−” indicates that the traffic is queued in the opposite direction to the driving direction [10].
**ff**	Free flow speed on the specific road segment	In kilometers/hour
**cn**	Confidence to the determined speed (indication of how the speed value was determined by the Here engine	−1.0 means the road is closed (for some time), 1.0 means 100% confidence (actual data from Here data sources). Historically inferred data are between 0.7 and 1
**jf**	Jam factor: expected quality of travel in terms of flow.	Values from 1 (free flow) to 10 (road closure). −1.0: value could not be calculated
**sp**	Speed, limited (capped) to by speed limit	In kilometers/hour
**su**	Speed, uncapped by the speed limit	In kilometers/hour, as determined by Here, represents the actual behaviour of the drivers: how fast they actually drive
**ty**	Type of information	Arbitrary string data
2. Raw XML files as provided by the Here Maps API web service. Each file corresponds to a unique city and a specific moment in time. These are stored into the **./xml.zip** archive and follow the naming structure **<cityName>_<day>-<month>-<year>_<hh><mm>.xml**, where **<hh>** and **<mm>** represent the local Bucharest time (GMT+2) (i.e. **timisoara_28-05-2020_1831.xml** is the file for city Timisoara, from 28th of May, 2020 at 18:31 local time).
3. Rendered images of the monitored road segments, with a discretization on four levels: from green (free flowing traffic) and up to brown (complete standstill). The images are following the **<cityName>_<day>-<month>-<year>_<hh><mm>.png** naming structure, where **<hh>** and **<mm>** represent the local Bucharest time (GMT+2). These images are used for qualitative validation of the data, by direct comparison with original rendering provided by the Here Maps Traffic and Google Traffic browser versions. The files are available as archives with the name **render.zip**, sorted in distinct folders, per city.

For a better interpretation of the flow data, stored in the .csv file, in Table 2 we provide a detailed description of the fields extracted from Here Maps Traffic API, with comments over the semantics and calculation of the fields. A more indepth documentation is to be found in [10,12].

For a more depth and complete analysis, taking into account the context of the data (the transportation and traffic restrictions imposed on the national level by the SARS-CoV-2/COVID19 pandemic) we present in Table 3 the most important events with impact over the traffic flow. These data can be augmented by the user of the dataset with supplementary data (such as weather), based on their own avenue of investigation.

**Table 3:** Most important administrative decisions and social events related to COVID19 restriction which might have impact on the road traffic flow.

Date	Event	Relevant impact over traffic flow
11-03-2020	Schools and Universities are closed for face-to-face activities	Morning commute reduced by lack of parents shutting kids to school
16-03-2020	State of Emergency imposed over territory of Romania	Prepares the context of imposing traffic restrictions
21-03-2020	Military Ordnance #2	Restricts movement from 22:00 to 06:00 to specific categories of users and reasons
24-03-2020	Military Ordnance #3	Completely restricts movements of citizens: any outside the home movement has to be backed by documents and checkpoints are organized on the public roads inside the city and on city boundaries
12-04-2020	Catholic Easter	
19-04-2020	Orthodox Easter	People are trying to go outside for Church celebration and/or for visiting families, traveling long distances with various “stated reasons”
15-05-2020	State of Alert	Downgrading the state of emergency to state of alert. Lifting of transportation restrictions over a 30km area around the center of metropolitan areas. Outside the 30km area documents for travel are still necessary
01-06-2020	Lifting of transportation restrictions	There is no need for any documents when travelling inside national borders.

**Fig. 1:**
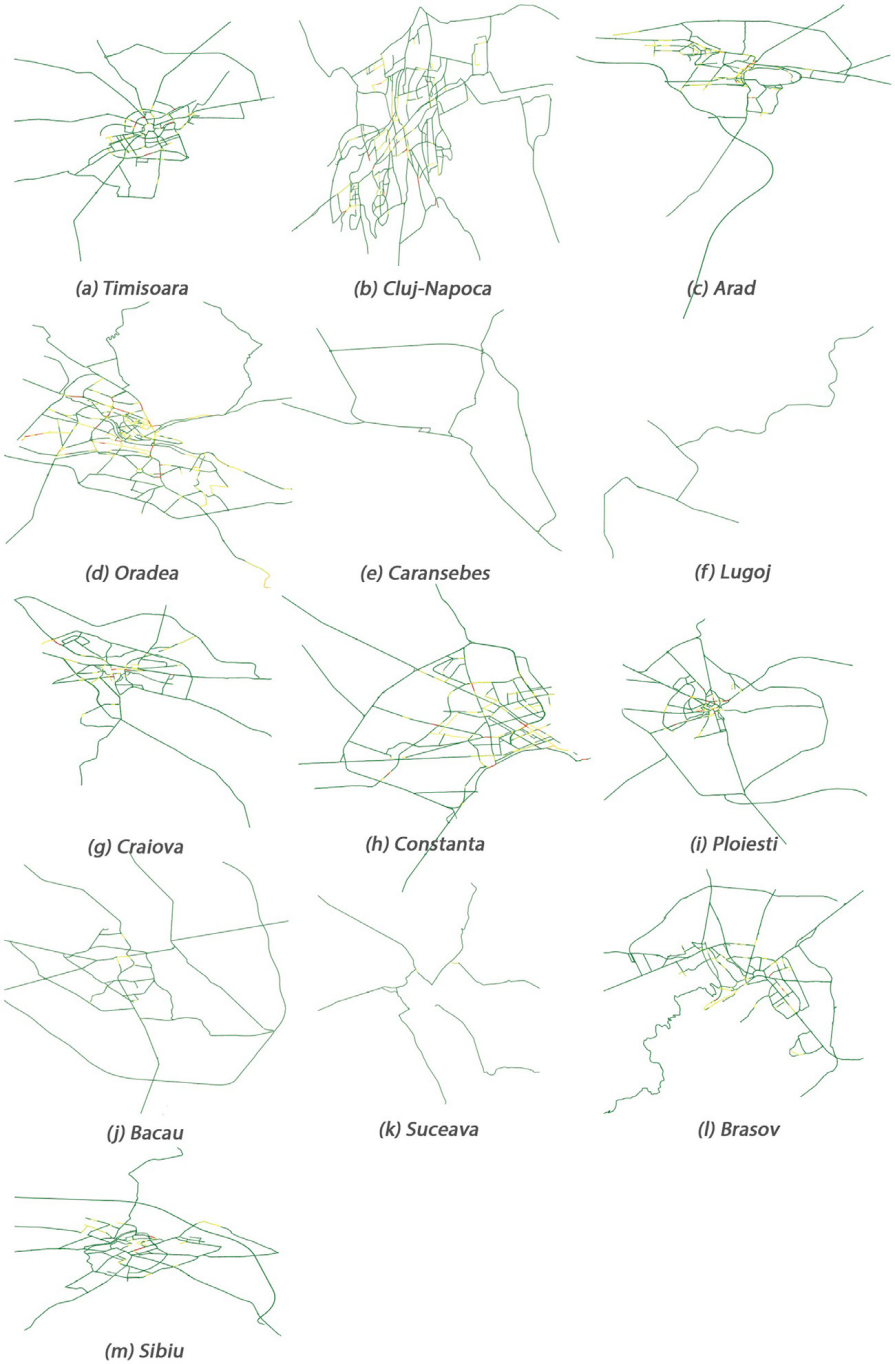
Snapshot of all the 13 cities which are part of the dataset at 1st. of June 2020, 12:31 local Romanian time showing the extent of the monitored areas (bounding boxes) and typical rendering on 4 discretization levels of the Jam Factor (JF).

**Fig. 2:**
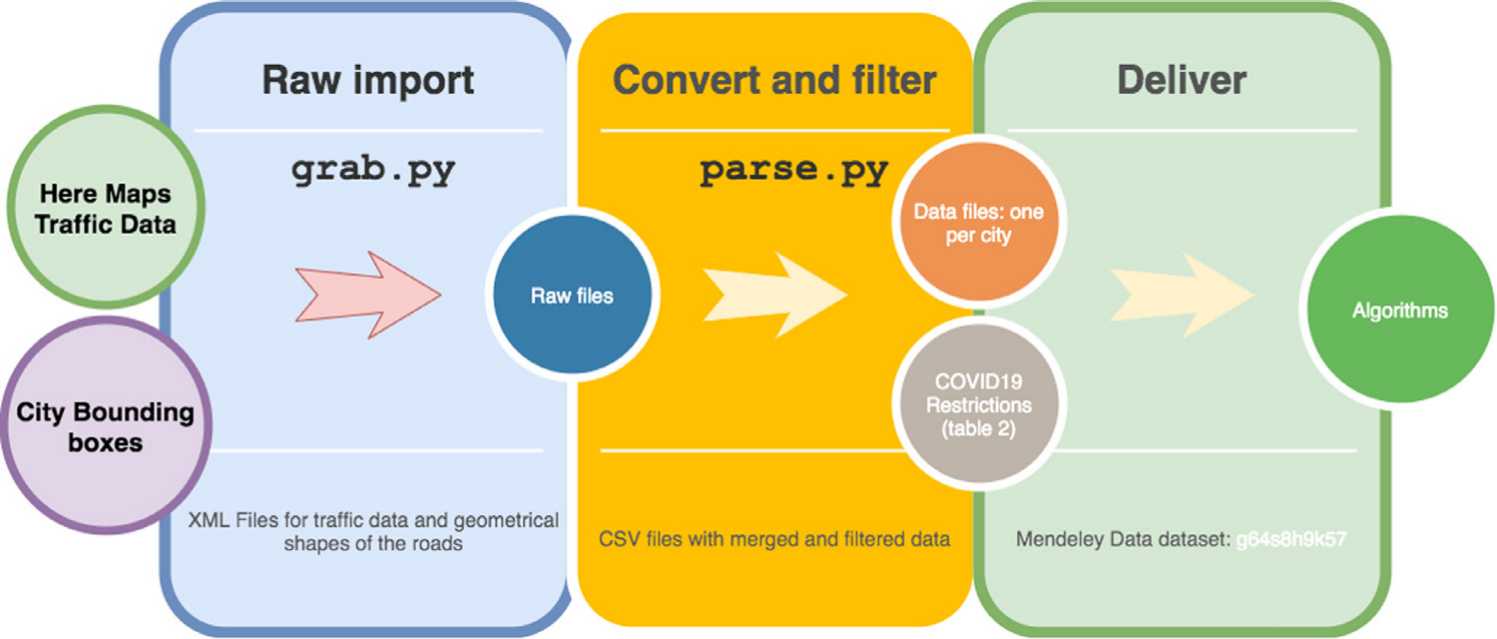
Workflow detailing the collection and processing of the data.

## 2 Experimental Design, Materials and Methods

**Fig. 3:**
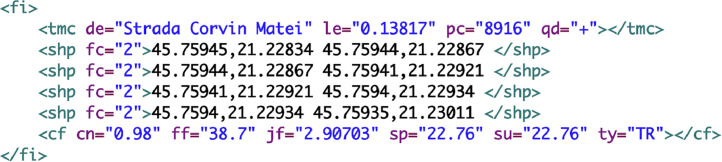
Typical data from Here Maps API: each flow item (fi) consists of TMC, flow data and geometrical data.

The data collection is done by a dedicated Python script, adapted from the one available at [11]. Using the Here Maps Traffic Flow API we query the web service for the data regarding each of the cities, defined by the bounding boxes presented in Table 1. For each of the queries, we get a list of **<fi>** items as an XML formatted response. The structure of each **<fi>** item, as exemplified by the record from Figure 3, consists of a **<tmc>** field which describes the static structural characteristics of the road, a list of shapes **<shp>** describing the road segments the geographical coordinates of the start and stop points of each segment, together with its *functional class* and a **<cf>** field with traffic flow related information.

The Python script, with detailed comments is available in the data repository under the name **grab.py**. The Here Maps API keys were removed and should be replaced by the user's keys.

The next level out automation is provided by the UNIX *cron* tool (but can be also implemented by Microsoft Windows Scheduled Tasks) and consists of a shell script for calling the grabbing script for each of the cities which need to be monitored. This file is available in the repository under the name **auto.sh**.

The output of the automated running of the **grab.py** script is represented by a set of files, grouped in folders by city name. Using the raw XML data we generate a **.png** image for each run with road segments colored according to the jam-factor in four classes of jamming, from green (almost free flow) and up to brown (complete standstill). These pictures are for qualitative evaluation of the data and are used for checking the correct functioning of the toolchain during long runs (weeks and months). The image data can be also used for visual displays of the quality of traffic as short movie clips. In this dataset, all the pictures are stored in the **./render.zip** archive, grouped per city name.

Further, the postprocessing of the data is done via the second Python script, **parse.py**, also available with the data repository. This one is called manually for each of the cities, passing as first command line arguments the **basePath** holding the XML files produced by multiple runs of grab.py. The second command-line argument is the label of the city (city name) and the third argument is represented by the base folder path for the output (where the post-processed CSV is to be stored).

For each of the XML files found into the **basePath**, the script is extracting the metadata encoded into the file-name (city, date and time) and iterates over the **<fi>** items extracting the relevant information for traffic flow. The structure of the data is described in the *Data Description* section. For defensive programming reasons checking of None type is done and default values are stored whenever the actual data are corrupted or missing (i.e. “DE” field representing the street/road name is missing and is replaced by “N/A”). For each folder (set of records about a specific city) the parse.py produces a concatenated **.csv** file with all the records available, one per line. These files, for each of the cities, represent the core element of this dataset and are provided distinctly per city, or as an archive with all the cities and all the records, in the data repository. The data regarding the shapes of the roads are discarded in the CSV files but are available in the raw XML files, stored under the **./raw** path in the dataset.

## Ethics Statement

This work did not include any human subjects nor animal experiments.

## Declaration of Competing Interest

The authors declare that they have no known competing financial interests or personal relationships which have, or could be perceived to have, influenced the work reported in this article.

## Appendix Supplementary materials

Image, application 1
